# Impaired Glucose Tolerance or Newly Diagnosed Diabetes Mellitus Diagnosed during Admission Adversely Affects Prognosis after Myocardial Infarction: An Observational Study

**DOI:** 10.1371/journal.pone.0142045

**Published:** 2015-11-16

**Authors:** Anish George, Raghav T. Bhatia, Gill L. Buchanan, Anne Whiteside, Robert S. Moisey, Stephen F. Beer, Sudipta Chattopadhyay, Thozhukat Sathyapalan, Joseph John

**Affiliations:** 1 Department of Cardiology, Scunthorpe General Hospital, Scunthorpe, United Kingdom; 2 Department of Cardiology, Castle Hill Hospital, Kingston upon Hull, United Kingdom; 3 Department of Diabetes and Endocrinology, Scunthorpe General Hospital, Scunthorpe, United Kingdom; 4 Department of Diabetes and Endocrinology, Calderdale and Huddersfield NHS Foundation Trust, Huddersfield, United Kingdom; 5 Department of Academic Endocrinology, Diabetes and Metabolism, Hull York Medical School, University of Hull, Kingston upon Hull, United Kingdom; Osaka University Graduate School of Medicine, JAPAN

## Abstract

**Objective:**

To investigate the prognostic effect of newly diagnosed diabetes mellitus (NDM) and impaired glucose tolerance (IGT) post myocardial infarction (MI).

**Research Design and Methods:**

Retrospective cohort study of 768 patients without preexisting diabetes mellitus post-MI at one centre in Yorkshire between November 2005 and October 2008. Patients were categorised as normal glucose tolerance (NGT n = 337), IGT (n = 279) and NDM (n = 152) on pre- discharge oral glucose tolerance test (OGTT). Primary end-point was the first occurrence of major adverse cardiovascular events (MACE) including cardiovascular death, non-fatal MI, severe heart failure (HF) or non-haemorrhagic stroke. Secondary end-points were all cause mortality and individual components of MACE.

**Results:**

Prevalence of NGT, impaired fasting glucose (IFG), IGT and NDM changed from 90%, 6%, 0% and 4% on fasting plasma glucose (FPG) to 43%, 1%, 36% and 20% respectively after OGTT. 102 deaths from all causes (79 as first events of which 46 were cardiovascular), 95 non fatal MI, 18 HF and 9 non haemorrhagic strokes occurred during 47.2 ± 9.4 months follow up. Event free survival was lower in IGT and NDM groups. IGT (HR 1.54, 95% CI: 1.06–2.24, p = 0.024) and NDM (HR 2.15, 95% CI: 1.42–3.24, p = 0.003) independently predicted MACE free survival. IGT and NDM also independently predicted incidence of MACE. NDM but not IGT increased the risk of secondary end-points.

**Conclusion:**

Presence of IGT and NDM in patients presenting post-MI, identified using OGTT, is associated with increased incidence of MACE and is associated with adverse outcomes despite adequate secondary prevention.

## Introduction

Newly diagnosed diabetes mellitus (NDM) and impaired glucose tolerance (IGT) diagnosed on pre-discharge oral glucose tolerance test (OGTT) are common in patients with myocardial infarction (MI) [1–3] and coronary artery disease (CAD) [4]. Current evidence suggests worse post- MI prognosis in both patients with preexisting diabetes mellitus and pre-diabetic states diagnosed on elevated admission blood glucose [5–10] and fasting blood glucose [11–14].

The effect of post challenge hyperglycaemia on major adverse cardiovascular events (MACE) after MI is uncertain. Abnormal glucose tolerance (AGT) in some studies [15, 16], but only NDM in others [17, 18] increased the risk of MACE. NDM but neither impaired fasting glucose (IFG) nor IGT in patients with CAD was associated with an increased MACE in the Euro Heart Survey on diabetes and the heart [4]. In patients with ST-elevation MI (STEMI) treated with primary percutaneous coronary intervention (PPCI) OGTT determined glucometabolic status does not seem to independently affect prognosis [19, 20]. Studies suggesting the adverse effect of newly diagnosed abnormal glucometabolic state on post-MI prognosis [15–18] recruited small number of patients before the widespread use of dual anti-platelet therapy, statin and drug eluting stent and did not clarify the independent effect of IGT or 2 hour post-load glucose on prognosis or test the relative ability of fasting and 2 hour post-load glucose in predicting prognosis.

In our study, we aspire to bridge this gap in the evidence base- by analysing data collected on patients admitted with MI who underwent pre-discharge OGTT. We aim to evaluate the relationship between their glucometabolic status and long-term prognosis.

## Research Design and Methods

### Patient cohort

After the GAMI study [1,15] and according to guidelines [21, 22, 23] all patients without preexisting diabetes mellitus admitted to our unit with a confirmed MI underwent pre-discharge OGTT as part of routine clinical care. This observational study includes all consecutive patients admitted to our unit between November 2005 and October 2008 who underwent OGTT and were prospectively followed up. Data on demographics, risk factors for CAD, history of confirmed CAD, pre-hospital and discharge medications, troponin I levels, and revascularisation status of every patient was prospectively entered into a local database for contribution to the Myocardial Infarction National Audit Project. Patients who died before the OGTT, were admitted under the surgeons, transferred to another centre for urgent revascularisation or did not tolerate the glucose drink for the OGTT were excluded. Mortality data was collected from the hospital care records for patients who died in hospital. For patients who died in the community, mortality data was obtained from the general practitioner medical records and confirmed by the data provided by the office of public health intelligence. As this study retrospectively reported on routinely collected anonymised data on standard clinical practice to contribute to a National Audit database, the East Yorkshire and North Lincolnshire Research Ethics Committee confirmed that formal patient consent and ethical approval was not required. We registered this study as an audit with the Local Audit and Clinical Governance Department.

On or after the third day of admission, venous plasma glucose was measured after an overnight fast of ≥8 hours (FPG). A standardised OGTT (venous plasma glucose measured 2 hours after administration of 75g glucose (2-h PG) in 200 ml water) was performed on the same day. Plasma glucose was enzymatically determined using the glucose oxidase method. Intravenous glucose solutions were not allowed, but anti-adrenergic agents were used if clinically indicated. Patients, who were clinically too unstable for the test on day 3, were tested later during that admission. All patients diagnosed with IGT and NDM were advised lifestyle modification including diet, physical activity and referred to the diabetologists for appropriate management that was overseen by them as out-patients.

## Definitions

MI was diagnosed according to the universal definition [24]. A smoker was a person currently smoking or had stopped for <2 years, hypertensive if treated for or newly diagnosed with hypertension, hypercholesterolaemic if total cholesterol >5 mmol/l or treated for it. History of CAD included a history of confirmed MI and/ or PCI or coronary artery bypass grafting. The glucometabolic state was classified as follows: normal glucose tolerance (NGT): FPG <6.1 mmol/l and a 2-h PG <7.8 mmol/l; imapired fasting glucose (IFG): FPG 6.1–6.9 mmol/l and 2-h PG <7.8 mmol/l; IGT: FPG <7 mmol/l and 2-h PG 7.8–11 mmol/l. NDM: FPG ≥7.0 and/or 2-h PG ≥11.1 mmol/l. AGT was defined as patients with IGT or NDM. Patients with a clinical diagnosis of diabetes mellitus prior to the index admission were excluded. Patients were classified as having diabetes on the basis of history, regardless of duration of disease or need for anti-diabetic agents. The diagnosis was established if the patient had been informed of the diagnosis by a physician before the admission or was taking oral hypoglycaemic agents, insulin, or receiving dietary therapy. Although current guidelines supports the use of glycosylated haemoglobin (HbA1c) in diagnosing diabetes, for the purpose of this study it was not incorporated into the methodology since it was not recommended in contemporary guidance and was not available for routine use at our center at the time of the study [21, 25, 26, 27].

### Outcome measures

All participants were followed up for a median of 48 months for outcomes. Follow-up data was complete for all patients. Completeness of follow up was ensured by manual review of hospital and general practice records. Importantly each event was recorded at the first occurrence only. Cardiovascular death was defined as death from myocardial infarction, heart failure, non-haemorrhagic stroke and sudden death without any obvious reason. A non-fatal re-infarction was defined as a non-fatal MI occurring later than 72 h after the index infarction. Stroke was defined as a neurological deficit persisting >24 hours as observed by a physician with radiological confirmation of haemorrhagic or non-haemorrhagic stroke. Severe heart failure (HF) was recognised when causing hospital admission requiring intensification of or additional treatment. The primary end-point was a composite outcome defined as the first occurrence of a MACE including cardiovascular death, non-fatal re-infarction, severe heart failure or non-haemorrhagic stroke. Secondary end-points were all cause mortality, cardiovascular mortality, non-fatal re-infarctions, severe heart failure or stroke.

### Statistical analysis

Continuous variables are presented as medians (inter-quartile range) and categorical variables as counts and proportions (%). Differences in baseline characteristics between the three groups were compared using the one-way analysis of variance and Kruskal-Wallis test for parametric and non-parametric data respectively for continuous variables and chi-squared test for categorical variables. Event free survival was estimated in the three glucometabolic categories from Kaplan–Meier curves that were compared using the Log-rank test. Cox proportional-hazards regression modeling was used to analyse the effect of several variables on event free survival. All covariates known to affect prognosis after MI including age, gender, smoking status, hypercholesterolaemia, hypertension, history of previous acute myocardial infarction, diagnosis at discharge, discharge prescription of aspirin, clopidogrel, beta-blockers, angiotensin-converting enzyme inhibitors and statins, revascularisation status and glucometabolic status were “entered” into the model. Results are reported as hazard ratios (HRs) with associated 95% confidence intervals (CIs). Multivariate logistic regression models were used to calculate odds ratios (ORs) and 95% confidence intervals (CIs) for events in each glucometabolic categories after adjustment for all the other clinical characteristics.

## Results

### Patients

About 90% of the 1489 index admissions with raised troponin between November 2005 and October 2008 had a diagnosis of MI confirmed. Patients previously known to have diabetes (n = 289) were excluded. The baseline characteristics of 768 patients without preexisting diabetes mellitus with MI who had the OGTT are shown in Table 1. Patients with IGT and NDM were older but all other clinical characteristics were similar in the 3 groups. OGTT was done on day 3 in 63.3% patients, day 4–7 in 33.3% and rest later. Patients who had their OGTT on day 3 had similar FPG (5.28 ± 0.75 v 5.20 ± 0.89, p = 0.17) and 2h-PG (8.75 ± 3.07 v 8.76 ± 3.14, p = 0.99) to those done later. About 63% of STEMI and 64% of NSTEMI patients had OGTT done on day three. The OGTT was done later in admission in STEMI than in NSTEMI patients (4.07±1.67 days v 3.82±1.37 days, p = 0.02). FPG (5.32 ± 0.80 v 5.21 ± 0.81, p = 0.06) and 2h-PG (8.76 ± 3.08 v 8.76 ± 3.11, p = 0.99) were similar in patients with NSTEMI and STEMI respectively. About 76% of patients presenting with STEMI were thrombolysed of which about 23% needed rescue PCI. Another 2% had PPCI. The rest were ineligible for thrombolysis and were not reperfused. Of the STEMI patients that did not undergo revascularisation acutely, 29% had ischaemia driven revascularisation (PCI in 72%). All patients with NSTEMI had coronary angiogram and 41% were revascularised (92% had PCI). Overall 42% of patients were revascularised (PCI 37%, CABG 5%). About 36% and 7% patients with STEMI and 38% and 3% with NSTEMI underwent PCI and CABG respectively.

**Table 1:** Clinical characteristics of the study population.

	NGT(n = 337)	IGT(n = 279)	NDM(n = 152)	p
Age (years; median; IQR)	62(17)	68(18)	68(19)	<0.001
Male n (%)	236(70)	204(73)	109(72)	0.70
Current smoker	101(30.0)	96(34.4)	46(30.3)	0.46
Hypertension	122(36.2)	117(41.9)	59(38.8)	0.35
Hypercholesterolaemia	151(44.8)	134(48.0)	67(44.1)	0.65
Previous myocardial infarction	51(15.1)	59(21.1)	29(19.1)	0.15
Known IHD	85(25.2)	89(31.9)	50(32.9)	0.10
Family history	138(40.9)	115(41.2)	65(42.8)	0.93
Discharge diagnosis of NSTEMI	196(58.2)	159(57.0)	92(60.5)	0.78
Discharge medications				
Aspirin	308(91.4)	255(91.4)	142(93.4)	0.72
Clopidogrel	280(83.1)	222(79.6)	129(84.9)	0.33
Dual anti-platelet	265(78.6)	207(74.2)	121(79.6)	0.31
Beta-blocker	260(77.2)	211(75.6)	116(76.3)	0.90
ACEI/ARB	270(80.1)	236(84.6)	128(84.2)	0.29
Statin	325(96.4)	264(94.6)	142(93.4)	0.30
Revascularised	147(43.6)	118(42.3)	58(38.2)	0.52
Troponin I (μg/l; median; IQR)	2.8(13.2)	3.2(11.0)	3.0(15.5)	0.82
FBG (mmol/l; median; IQR)	4.9(0.5)	5.1(0.8)	5.7(1.3)	<0.001
2HBG (mmol/l; median; IQR)	6.3(1.6)	9.2(1.9)	12.9(2.7)	<0.001

FPG categorised 689 (89.7%) patients as NGT, 48 (6.3%) as IFG and 31 (4.0%) as NDM. After OGTT, 328 (42.7%) patients had NGT, 9(1.2%) had IFG, 279 (36.3%) had IGT and 152 (19.8%) were NDM. As the 9 IFG patients by definition did not have post-load hyperglycaemia, they were included in the NGT group for purposes of the analysis. Of the 689 patients diagnosed normal on FPG alone, 261 (37.9%) had IGT and 100 (14.5%) had NDM. Of the 48 patients diagnosed IFG on FPG alone, 17 (37.4%) had IGT and 22 (45.8%) had NDM. Of the 431 (56.1%) patients with post- load hyperglycaemia, 361 (83.8%) had normal FPG.

### Outcomes

Table 2 summarises all first events during 47.2 ± 9.4 months of follow up. A total of 224 events (102 deaths from all causes, 95 non fatal myocardial infarctions, 18 heart failure admissions and 9 non haemorrhagic strokes) occurred during follow up. Of the 102 deaths from all causes 32 (9.5%) with NGT, 44 (15.8%) with IGT and 26 (17.1%) with NDM), 79 occurred as the first event. A further 23 deaths occurred during follow up of patients who reached a non-fatal end-point. Of these 13 were cardiovascular deaths (3 in NGT, 8 in IGT and 2 in NDM groups), 4 cancer, 3 sepsis, 2 haemorrhagic strokes and 1 chronic obstructive pulmonary disease. Of the 33 non-cardiovascular deaths that that occurred as first event, 13 were due to cancer, 6 haemorrhagic stroke, 7 pneumonia, 3 sepsis, 2 acute renal failure, 1 gastrointestinal bleed and 1 chronic obstructive pulmonary disease.

**Table 2:** First cardiovascular events in relation to glucometabolic status. Events that occurred until death or Feb 1, 2011. Each event was recorded only once.

	NGT (n = 337)	IGT (n = 279)	NDM (n = 152)	p	Total (n = 768)
Death	24(7.1)	33(11.8)	22(14.5)	0.027	79(10.3)
Non-cardiovascular	13(3.9)	12(4.3)	8(5.3)	0.778	33(4.3)
Cardiovascular	11(3.3)	21(7.5)	14(9.2)	0.015	46(6.0)
Non-fatal re-infarction	31(9.2)	35(12.5)	29(19.1)	0.009	95(12.4)
Non-haemorrhagic stroke	1(0.3)	6(2.2)	2(1.3)	0.102	9(1.2)
Heart failure re-admission	5(1.5)	9(3.2)	4(2.6)	0.351	18(2.3)
MACE*	48(14.2)	71(25.5)	49(32.2)	0.000	168(21.8)

* Major adverse cardiovascular events: first occurrence of cardiovascular death, non- fatal re-infarction, non-haemorrhagic stroke or severe heart failure.

**Fig 1:**
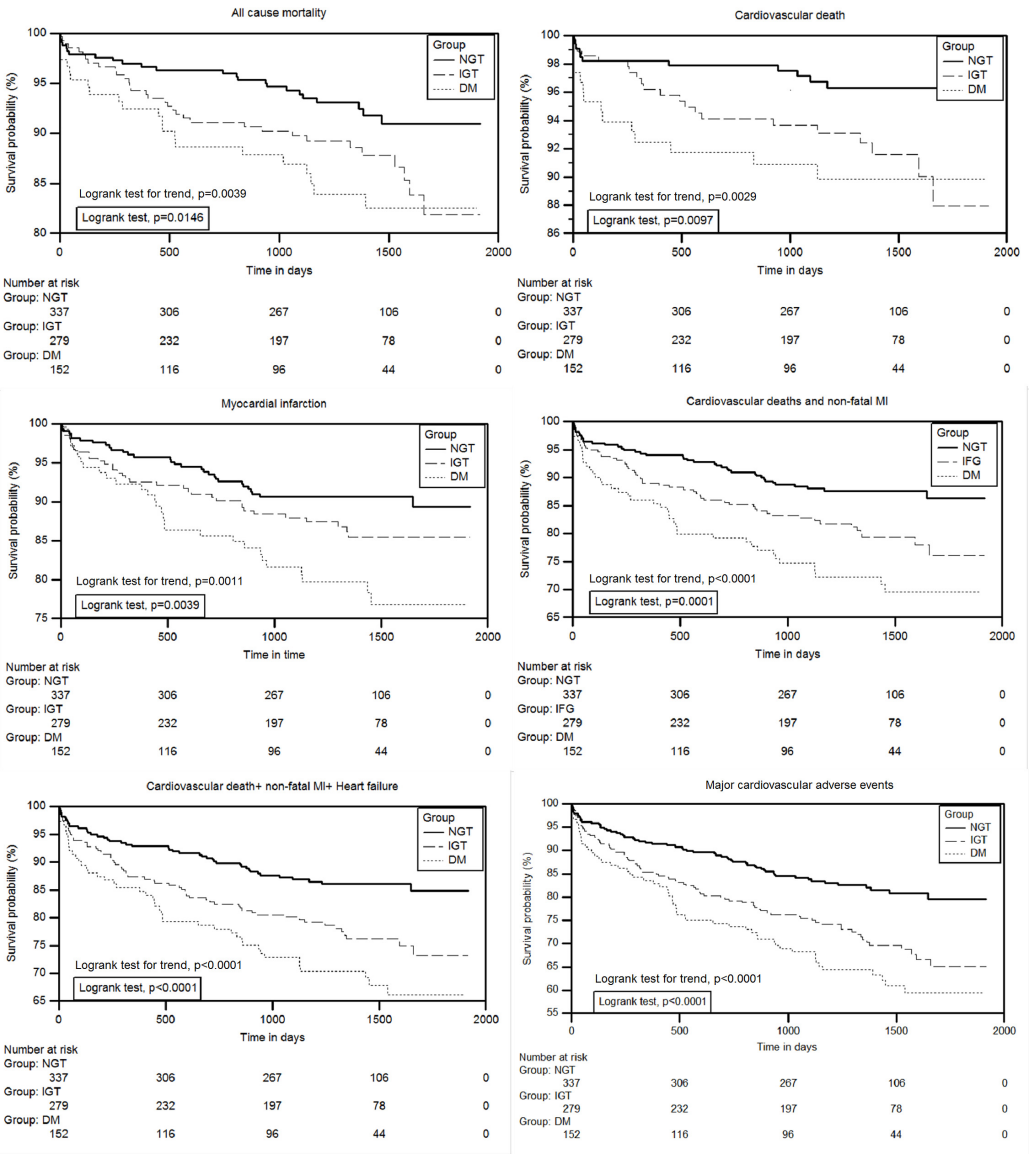
Kaplan–Meier estimates for the three groups showing survival free of all cause mortality. Numbers below graph are the number of patients at risk. Survival compared using log-rank test.

The incidence of adverse events progressively increased from the NGT to the NDM group (Table 2). Event-free survival was significantly different in the three groups (Fig 1). After adjustment for covariates using Cox proportional hazard modeling (Table 2), NDM and IGT were strong independent predictors of survival free of the primary end-point. NDM also independently predicted all cause mortality (HR 2.14, 95% CI: 1.17–3.94; p = 0.0145), cardiovascular mortality (HR 2.83, 95% CI: 1.24–6.45; p = 0.0135), non-fatal MI (HR 1.96, 95% CI: 1.16–3.29; p = 0.0121) and combined cardiovascular death or non-fatal MI (HR 2.17, 95% CI: 1.39–3.38; p = 0.0007). Absence of AGT predicted reduced cardiovascular deaths (HR 0.45, 95% CI: 0.23–0.91; p = 0.0257), combined cardiovascular death or non-fatal MI (HR 0.62, 95% CI: 0.43–0.90; p = 0.0125) and combined cardiovascular death or non-fatal MI or heart failure (HR 0.61, 95% CI: 0.43–0.87; p = 0.0069). The adjusted ORs for MACE were significantly higher in the IGT, NDM and AGT groups. The odds of the secondary end-points increased with NDM and AGT but not IGT (Table 3).

**Table 3:** Candidate predictors of major cardiovascular events free survival for the entire study population using Cox proportional hazard modelling.

Covariates	p	HR	95% CI
Age	<0.0001	1.037	1.02–1.05
Previous myocardial infarction	<0.0001	2.281	1.63–3.18
Newly diagnosed DM	0.0003	2.148	1.42–3.24
Newly diagnosed IGT	0.0242	1.543	1.06–2.24
Discharged without beta-blockers	0.0365	1.446	1.03–2.04
Discharge diagnosis of STEMI	0.0475	1.382	1.01–1.90
Discharged without ACEI	0.0614	1.442	0.98–2.11
Hypertension	0.1001	1.303	0.95–1.78
Discharged without Aspirin	0.1297	1.447	0.90–2.33
Revascularised	0.1407	1.273	0.92–1.75
Hypercholesterolaemia	0.3550	0.860	0.63–1.18
Discharged without clopidogrel	0.4246	1.162	0.80–1.68
Female gender	0.4765	0.879	0.62–1.25
Current smoker	0.6055	0.904	0.62–1.32
Discharged without statin	0.6460	1.152	0.63–2.10

Log-likelihood ratio tests were used to compare the fit of predictive models using categories based on FPG and OGTT only and in combination (Table 4). Comparing nested models showed that including the OGTT categories significantly improved the predictability of the model based on FPG categories and above noted risk factors, for MACE (χ2 = 26.41, df = 3, p = 0.00), cardiovascular death (χ2 = 9.1, df = 3, p = 0.03), non- fatal MI (χ2 = 11.75, df = 3, p = 0.01) and combined end-point of cardiovascular deaths and non-fatal MI (χ2 = 19.18, df = 3, p = 0.00). In contrast, the addition of categories based on FPG only to the model based on OGTT categories and risk factors did not significantly improve the prediction of the model for MACE (χ2 = 0.44, df = 2, p = 0.80), cardiovascular deaths (χ2 = 0.59, df = 2, p = 0.74), non-fatal MI (χ2 = 0.08, df = 2, p = 0.96) and combined end-point of cardiovascular deaths and non-fatal MI (χ2 = 0.13, df = 2, p = 0.94).

**Table 4:** Adjusted logistic regression for the primary and secondary end-points at the end of follow up according to glucometabolic state after the oral glucose tolerance test.

	NGT	IGT			DM		
		OR*(OR**)	95% CI	p	OR* (OR**)	95% CI	p
All death	1.00	1.28 (1.75)	0.69–2.40 (1.01–3.04)	0.436 (0.047)	2.05 (2.21)	0.99–4.28 (1.19–4.08)	0.055(0.011)
Cardiovascular death	1.00	1.83 (2.41)	0.81–4.09 (1.14–5.09)	0.144(0.021)	3.40 (3.01)	1.31–8.85 (1.33–6.79)	0.012(0.008)
Non-fatal MI	1.00	1.19 (1.42)	0.69–2.06 (0.85–2.36)	0.527 (0.183)	2.09 (2.33)	1.17–3.75 (1.35–4.03)	0.013(0.002)
CVS death or non-fatal MI	1.00	1.41(1.76)	0.87–2.28 (1.14–2.73)	0.159(0.011)	2.59 (2.77)	1.53–4.40 (1.72–4.47)	0.000(0.000)
CVS death, non-fatal MI or CCF	1.00	1.52(1.87)	0.96–2.40 (1.24–2.84)	0.071 (0.003)	2.52 (2.76)	1.51–4.20 (1.74–4.38)	0.000(0.000)
MACE	1.00	1.74 (2.05)	1.11–2.72 (1.36–3.08)	0.016 (0.000)	2.71 (2.86)	1.63–4.50 (1.81–4.52)	0.000(0.000)

* Odds ratio adjusted for age, gender, previous history of myocardial infarction, smoking status, hyperlipidaemia, hypertension, discharge diagnosis (STEMI, NSTEMI), discharge medications i.e. aspirin, clopidogrel, statin, ACEI or ARB and beta-blockers and revascularisation.

**Unadjusted Odds ratio.

## Conclusion

Our study confirms that (1) a large proportion of patients without preexisting diabetes mellitus admitted with MI have new DM and IGT that would remain undiagnosed on FPG only, (2) the incidence of MACE after MI progressively increases from the patients with NGT to NDM, (3) MACE-free survival in these patients is worse not only with NDM but also with IGT and (4) both NDM and IGT are strong predictors of MACE.

It is well recognized that the vascular risk associated with glucometabolic disorders appear early in the course of the disease spectrum, and years ahead of the development of diabetes [28]. The high prevalence rate of IGT in our study is in keeping with this and suggests that risk of MI extends to patients with pre-diabetic conditions as well.

This is by far the largest study assessing the effect of newly diagnosed AGT on prognosis after MI. Most previous studies [15–17] recruited fewer patients before the widespread use of statins, clopidogrel and drug eluting stents. The effect of IGT on prognosis remained unclear in previous studies. Although both the GAMI group [1, 15] and Tamita et al [16] suggest the independent effect of AGT on MACE, neither study report the adjusted hazard of MACE in patients with IGT and NDM separately. Thus it is unclear whether IGT affected prognosis in these studies. Kitada et al [17] reported similar MACE rates in the NGT and IGT groups with higher MACE rates in NDM. MACE predominantly consisted of revascularisation for restenosis and de novo lesions with very few cardiovascular events. Euro Heart Survey [4] reported prognosis in 2515 non-diabetic patients with CAD, included 1029 with MI classified based on OGTT. Within the whole population, NDM but not IGT independently increased the risk of death but not the other end-points. As MACE in the MI patients has not been published separately, it is impossible to infer the prognostic effect of the glucometabolic states in these patients from this study. Our study clearly demonstrates that not only NDM but also IGT independently adversely affects prognosis after MI. This study confirms previous reports [1, 16, 27, 29] that FPG alone underestimates the prevalence of abnormal glucometabolic state. About 90% of our patients were diagnosed normal by FPG only and 84% of patients with AGT had normal FPG. In the DECODE study, about 37% of NDM diagnosed on OGTT were normal on FPG criteria [27]. In the GAMI population [1], 10% had NDM on FPG criteria compared to 31% on OGTT criteria. The reliability of pre-discharge OGTT in reflecting “true” glucometabolic state may be dependent on its timing in relation to the cardiac event and the severity of the event itself. The pre-discharge glucometabolic category may [19, 20, 29] or may not [30, 31] change in the medium to long term in these patients. Glucometabolic categories based on OGTT done within 24 hours of a STEMI changed in 46% patients at 3 months post-discharge [29]. Glucometabolic abnormality seems to be overestimated within the first 24 hours after STEMI [19, 29]. However, OGTT done at or after 5 days seems to reliably predict long term glucometabolic state [30, 31]. This may be related to the subsidence of the acute responses between 2–5 days with no further decrease thereafter [31]. Hage et al [32] suggested a somewhat better reproducibility of OGTT in patients with subendocardial than transmural infarction. All our patients had the OGTT at least three days (37% ≥ day 4) after the index event. This makes it likely that the results would reliably predict long term glucometabolic state. Irrespective of its relation to long term glucometabolic status, pre-charge OGTT based classification independently predicted prognosis in our post-MI patients.

While the measurement of HbA1c would have improved the understanding of the glucometabolic status of these patients, especially with regard to the role of stress induced hyperglycaemia in the prevalence of abnormal glucose tolerance; this was not available for use at our center on a routine basis. Furthermore, while HbA1c identifies patients with NDM, it has limited use in the identification of patients with intermediate hyperglycaemia or IGT. Recent studies have shown an increased incidence of adverse events in non-diabetic post MI patients with elevated HbA1c, suggesting that the underlying mechanism is not entirely a stress mediated response [28]. Furthermore, the purpose of the study was to ascertain the prognostic risk of post challenge hyperglycaemia in patients following acute MI, irrespective of the underlying pathophysiology.

There are some differences in baseline characteristics across the three groups, which could have influenced the results. Patients with IGT and NDM were older than the patients in NGT group, which was noted to be statistically significant. Although we have adjusted for this in the cox proportional hazard regression model and while it is unlikely to explain the increased incidence of adverse events in the IGT and NDM group; it is difficult to be certain if there any residual confounding in the final result. Similar baseline variation, especially in age, between NGT and AGT has been noted in other similar studies as well [15, 19].

The MACE rates in our study are similar to those previously reported [15–17, 19]. Contrary to some [16, 17], our study suggests that clinical events rather non-target vessel revascularisation may predominate MACE in post-MI patients newly diagnosed with IGT or DM. The management of our patients matched contemporary published data [4, 15]. The revascularisation rate was similar to the GRACE registry [32]. Compared to the EHS-ACS II survey [33], PCI rates were lower in STEMI, as most patients were thrombolysed, but similar in NSTEMI patients.

In the absence of robust evidence for prognostic, benefits of intensive glycaemic control [34], none of our patients underwent in- hospital intensive glucose lowering therapy. They were not discharged on hypoglycaemic agents due to the uncertainty whether the dysglycaemia detected pre-discharge truly reflected the “true” glucometabolic status. All IGT and NDM patients were advised lifestyle modification including diet, physical activity and were referred to the cardiac rehabilitation service. They were also referred to the diabetologists for appropriate management that was overseen by them as out-patients.

## Limitations

Being an observational longitudinal cohort study using retrospective analysis of prospectively collected data from a single centre, it has its limitations. In line with all other studies [4, 15– 17], we did not explore the interaction between either acute revascularisation and post-challenge hyperglycaemia or post-discharge management of the glucometabolic status and prognosis. As OGTT was not repeated pre- or post- discharge, it is uncertain whether random fluctuation in glycaemia or stress associated hyperglycaemia affected results. However, as pre-discharge post-challenge hyperglycaemia, irrespective of its pathophysiological mechanism, diagnosed on these measurements predicted outcomes in post-MI patients, the reproducibility of these measurements and its relation to long term glucometabolic status may be less relevant when assessing prognostic risk. Though we could not include a small proportion of non-diabetic post-MI patients admitted to our institution who could not have OGTT for valid reasons, we feel that it would be acceptable to extend the results of this study to the whole population.

In summary, our study suggests for the first time that risk of MACE after an MI is increased not only in newly diagnosed DM but also in IGT. It provides further evidence to support the previous finding that dysglycaemia is common post MI in patients without preexisting diabetes mellitus. Importantly as the glucometabolic status of a large proportion of patients would be misdiagnosed on FPG only, an appropriately timed OGTT after MI, may be helpful in assessing prognostic risk and plan future management.
